# Role of Collagen Fiber Morphology on Ovarian Cancer Cell Migration Using Image-Based Models of the Extracellular Matrix

**DOI:** 10.3390/cancers12061390

**Published:** 2020-05-28

**Authors:** Samuel Alkmin, Rebecca Brodziski, Haleigh Simon, Daniel Hinton, Randall H. Goldsmith, Manish Patankar, Paul J. Campagnola

**Affiliations:** 1 Department of Biomedical Engineering, University of Wisconsin-Madison, Madison, WI 53706, USA; alkmin@wisc.edu (S.A.); brodziski@wisc.edu (R.B.); hsimon3@wisc.edu (H.S.); 2 Department of Chemistry, University of Wisconsin-Madison, Madison, WI 53706, USA; dhinton@chem.wisc.edu (D.H.); rhg@chem.wisc.edu (R.H.G.); 3 Department of Obstetrics and Gynecology, University of Wisconsin-Madison, Madison, WI 53706, USA; patankar@wisc.edu

**Keywords:** collagen, ovarian stroma, motility, Second-Harmonic Generation, multiphoton excited, cytoskeleton, cadherin

## Abstract

Remodeling of the extracellular matrix (ECM) is an important part in the development and progression of many epithelial cancers. However, the biological significance of collagen alterations in ovarian cancer has not been well established. Here we investigated the role of collagen fiber morphology on cancer cell migration using tissue engineered scaffolds based on high-resolution Second-Harmonic Generation (SHG) images of ovarian tumors. The collagen-based scaffolds are fabricated by multiphoton excited (MPE) polymerization, which is a freeform 3D method affording submicron resolution feature sizes (~0.5 µm). This capability allows the replication of the collagen fiber architecture, where we constructed models representing normal stroma, high-risk tissue, benign tumors, and high-grade tumors. These were seeded with normal and ovarian cancer cell lines to investigate the separate roles of the cell type and matrix morphology on migration dynamics. The primary finding is that key cell–matrix interactions such as motility, cell spreading, f-actin alignment, focal adhesion, and cadherin expression are mainly determined by the collagen fiber morphology to a larger extent than the initial cell type. Moreover, we found these aspects were all enhanced for cells on the highly aligned, high-grade tumor model. Conversely, the weakest corresponding responses were observed on the more random mesh-like normal stromal matrix, with the partially aligned benign tumor and high-risk models demonstrating intermediate behavior. These results are all consistent with a contact guidance mechanism. These models cannot be synthesized by other conventional fabrication methods, and we suggest this approach will enable a variety of studies in cancer biology.

## 1. Introduction

According to the American Cancer Society, ovarian cancer ranks fifth in cancer deaths among women in the United States. If detected in early stages, the five-year relative survival rate is 92% [1]; however, less than 20% of the cases are detected at a localized stage [2]. This is due to both the prevalence of non-specific symptoms and also the lack of early sensitive and specific screening/imaging methods to detect small tumors before they become metastatic [3,4,5,6,7]. This is an especially critical problem for ovarian cancer, as the primary metastatic mechanism is exfoliation from the surface epithelium to the peritoneum, which can occur during early disease stage [8,9,10,11]. Importantly, the five-year survival rate for high grade metastatic disease is ~25%.

A better understanding of the composition in the tumor microenvironment (TME) in this cancer could potentially lead to development of effective biomarkers and new therapies [12,13,14]. For example, while the dynamic interplay between cells and the extracellular matrix (ECM) influences differentiation, proliferation, and migration in both normal and tumor cells [12], it is not well understood how tumor growth depends on alterations in the matrix composition or morphology. Specifically, while migration is a hallmark of all cancers, it has not been well studied in ovarian cancer with respect to ECM remodeling. The lack of mouse models that represent human disease further complicates this problem, where these have been mainly limited to xenografts [15]. New mouse lines have been developed with specific mutations representing human disease, but their in vivo use lies in the form of implantation and monitoring subsequent tumor growth [16,17]. Thus, while promising, this application represents metastasis rather than primary disease in the fallopian tubes or ovary. There remains a clear need for biomimetic in vitro models that represent stromal alterations of the ovarian TME that allow hypothesis testing of the roles of the tumor cells and ECM morphology in disease progression.

A necessary first step to create such models is accurately characterizing the underlying stromal changes. The majority of the normal ovarian stroma comprises collagen type I with nonspecific fiber orientation/alignment, other minor matrix proteins, and stromal cells (e.g., fibroblasts and myofibroblasts) [18]. Importantly, alterations in Col I architecture and loss of ECM components (e.g., collagen IV) have been associated with ovarian carcinogenesis, where these likely extend throughout disease progression [19,20]. Additional changes in other components including fibronectin and laminin have also be documented [19].

Our lab has focused on examining changes in the collagen I stromal architecture using Second-Harmonic Generation (SHG) imaging microscopy. This 3D modality has great specificity/sensitivity for visualizing changes in fiber morphology as well as extracting underlying aspects of collagen architecture, for example average fibril size and macro/supramolecular helical attributes [21,22]. We have specifically examined such changes in a spectrum of human ovarian tumors and used these alterations as components in classification schemes [23,24,25,26]. For example, we have shown that six classes of ovarian tissues (e.g., normal and tumors) can be quantitatively differentiated using machine learning techniques based on the respective collagen morphology [24,25]. An overarching conclusion of all our studies is that the remodeling in high-grade serous ovarian cancer (HGSOC) is in the form of newly and improperly made collagen, creating a reactive stroma. We have further showed that these alterations are mostly present in the first 200 µm below the surface epithelium where the collagen is the most dense [26]. These observations form the rationale for creating tissue engineered scaffolds of the Col I architecture near the surface epithelium to study the corresponding cell biology. This is further supported by the fact that the large majority (~85%) of human ovarian cancers arise from the surface epithelium [27,28,29,30], and that the primary metastasis mechanism is exfoliation from the surface to the intra-peritoneal cavity.

Collectively, these observations lead to questions of the role of the collagen alterations on migration, cytoskeletal dynamics, and proliferation. However, there has been a lack of in vitro cancer models that can incorporate the native collagen fiber morphology in ovarian and other cancers. For example, self-assembled gel models have been successfully used to demonstrate the influence of collagen fiber size and density on migration persistence in breast carcinomas [31]. We have also used analogous models to examine the role of different collagen isoforms (Col III and V) on collagen assembly [22,32]. However, these gels have limited control in terms of fiber length, alignment, and spacing, resulting in models that poorly replicate the topography of the stromal microenvironments in different classes of tissues. Soft and hard photolithographies provide more control in terms of spatial resolution; however, the use of masks limits the replication of complex 3D collagen structures. Flow chambers and microfluidic approaches have identified important ECM species involved in migration and extravasation dynamics, but these models cannot replicate the in vivo collagen morphology [33,34,35,36].

To address these limitations, we developed a microscope-based system that utilizes multiphoton excited (MPE) photochemistry to synthesize in vitro biomimetic models [37,38,39,40,41]. The method is akin to 3D printing but produces submicron feature sizes and can utilize collagen and its analogs to reproduce the complex fiber morphology of the ovarian ECM. The fabrication resolution, or minimum feature size, is about 0.5 µm in diameter, which makes it a powerful tool to reproduce the native fiber widths and lengths [42]. We have previously shown that simple MPE fabricated ECM patterns (e.g., collagen IV, laminin, and fibronectin), and concentration gradients thereof, govern cell migration dynamics of different cell types, including breast and ovarian cancer, fibroblasts, and mesenchymal stem cells [43,44,45,46].

More recently, we introduced an image-inspired approach to study normal ovarian epithelial IOSE cells on scaffolds representing human tissues and simplified models thereof [46]. Specifically, we examined migration dynamics by decoupling fiber shape and fiber alignment and found that, while both contributed to the overall response, the highly periodic fiber shape in HGSOC greatly promoted motility. We now extend that study to compare the response of ovarian cancer cells on image-based scaffolds, where we also analyze additional markers and begin a mechanistic study to decouple the respective cell and matrix contribution to migration dynamics. These studies may identify new diagnostic/prognostic targets based on the collagen fiber structure.

## 2. Methods and Materials

### 2.1. Microscope and Photochemistry

The fabrication instrument was a purpose-built multiphoton microscope and has been described in detail previously [47]. A mode-locked titanium sapphire femtosecond laser (Mira; Coherent, Santa Barbara, CA, USA) was integrated with an upright microscope stand (Axioskop 2, Zeiss, Thornwood, NY, USA). Scanning was performed by two galvo mirrors (Cambridge Technologies, Bedford, MA, USA) for individual fields of view, which were then tiled together by a 3D motorized stage (Ludl Electronic Products Ltd., Hawthorne, NY, USA) to fabricate larger structures for cell analysis. A wavelength of 740 nm was used for two-photon excitation of the photo-initiator (see below), with approximately 100 mW average power at the plane of focus, where this was controlled by a 10 KHz electro-optic modulator (EOM, Conoptics, Danbury, CT, USA). We previously showed that the minimum feature sizes for crosslinked protein agreed with the theoretical resolution. For example, using 0.75 NA and 740 nm two-photon excitation, the respective lateral and axial resolutions were ~600 nm and 1.8 µm [42].

A key technology challenge in creating the stromal models is developing the scan control mechanism that can match the relative collagen concentration between each pixel in the image to that in the fabricated construct. Due to the inertia of the scanning mirrors, random scanning is not an optimal method of controlling the laser exposure. Instead, we implemented an approach we termed modulated raster scanning. Here the galvo mirrors were raster scanned (as in conventional or multiphoton microscopy), and the laser was rapidly shuttered by a second higher-speed EOM (maximum 100 MHz, Conoptics). The “open” fraction in each pixel (~10 microseconds) was mapped to the grayscale level (bits) of the corresponding pixel in the original SHG image. Thus, increased laser exposure linearly corresponds to in increased crosslinked collagen concentration [47].

We used the water-soluble Irgacure Sodium 4-[2-(4-morpholino)benzoyl-2 dimethylamino]butylbenzenesulfonate (MBS) as the photo-initiator. This moiety is non-toxic and has comparable efficiency to conventional vinyl photo-initiators soluble in organic solvents. MBS was synthesized in house using published protocols [48], where the properties were validated through standard spectroscopy characterization. Two-photon excitation of MBS (1 nM) at 740 nm drives a photochemical reaction that makes benzoyl and alpha amino alkyl radicals. At the focal volume, reactive radicals interact with the collagen and collagen analogs (described below), where the resulting radicals attack a second molecule inducing a chain crosslinking reaction and eventual termination [49].

### 2.2. Sample Preparation

For all scaffolds, we utilized a combination of solubilized 80% gelatin methacrylate (GelMA) + 20% Col I (150 mg/mL GelMA + 10 mg/mL type I collagen) as the starting material. GelMA was prepared from well-established protocols without further modification [50]. While it would be ideal to use all collagen I, solubility and pH issues make this approach difficult. GelMA is often used as a collagen substitute in tissue engineered scaffolds as it is biomimetic [51,52], and its use makes the photochemistry more facile. While the single-stranded GelMA presents RGD cues, GFOGER is the relevant binding site in the collagen triple helix, and the GelMA/collagen mixture then offers both the structural integrity and proper binding cues of the former and latter, respectively.

The substrates for the scaffolds were prepared first with a rubber hybridization chamber (~200 micron volume) secured to a silanized microscope slide [43]. To prevent non-specific adsorption, a monolayer of 30 mg/mL bovine serum albumin (BSA) was formed on the slide before the fabrication solution (MBS and GelMA/collagen) was applied [43]. The solution was kept strictly below 40 °C to avoid self-polymerization. Scaffolds were kept in phosphate-buffered saline (PBS) until cell seeding. Following the fabrication, un-crosslinked starting materials were dissolved away in a water bath at 37 °C.

### 2.3. Cell Seeding and Time-Lapse Imaging

Three ovarian epithelial cell lines of varying characteristics were used in this study: HEY (highly metastatic; from Dr. Molly Brewer, UCONN Health Center), OVCA433 (moderately metastatic; co-author Dr. Manish Patankar, WI, USA), and Immortalized Ovarian Surface Epithelium (IOSE; from Dr. Molly Brewer, MD, UCONN Health Center) as the normal control [53,54]. These three cell lines were cultured at 37 °C with 5% CO_2_ in DMEM/F12 medium base (LifeTechnologies 11330, Carlsbad, CA, USA) supplemented with 10% FBS (LifeTechnologies 10082).

Following fabrication and prior to cell seeding, the scaffolds were sterilized with 1X PBS containing 100 U/mL penicillin–streptomycin (Invitrogen 15140-122, Carlsbad, CA, USA). All cell lines were seeded at a density of 50K cell/mL and incubated overnight. Time-lapse studies were then performed by phase-contrast imaging (Nikon Ti-Eclipse inverted microscope with Pathology Devices, Inc., LiveCell^TM^ incubator system). Phase-contrast images (10×, 0.25NA objective) of each seeded scaffold were collected at 30 min intervals over 72 h. Cells became too densely populated at longer times to isolate the cell–matrix interactions, and collective migration events were not tracked. At least three independent measurements were used for each cell/scaffold combination with 60–80 attached cells in each case.

### 2.4. Cell Tracking

Tracking of cell migration was performed with Imaris (v7.6.5, Bitplane AG). For each fabricated scaffold, at least 20 cells were tracked for statistical significance. The resulting trajectory was directly exported to a spreadsheet and then analyzed in self-written code in MATLAB (Mathworks, Natick MA). This code outputs (i) cell position, (ii) the instantaneous speed, (iii) direction of the migration, and (iv) mean square displacement (MSD; $d^{2}\left(t\right)$). Motility coefficients (µ) were then determined by applying non-linear least-squares regression modeling of the MSD measurement to [55]:

(1) $$\langle d^{2}\left(t\right)\rangle =2n_{d}\mu \left[t-P(1-e^{-\frac{t}{p}})\right]$$

where *P* is the directional persistence time, and $n_{d}$ is the dimensionality and equals 2 here. Cell shape characteristics (spread area, circularity) were determined with ImageJ software.

### 2.5. F-Actin, Focal Adhesion, and Cadherin Staining

The ovarian cells were grown on the scaffolds between 16 and 24 h prior to staining for actin stress fibers, focal adhesions, and N/E-cadherin. For actin staining, the cells were fixed with 4% paraformaldahyde in PBS for 15 min. Following two washes with 1× PBS, the cells were permeabilized with 0.3% Triton X-100 for 10 min and stained with Texas Red conjugated phalloidin for 30 min. Two-photon excited fluorescence images were collected using a 40× 0.8NA objective. This was done for both IOSE and OVCA433 cells, with cells analyzed for each scaffold. CurveAlign [56] was used to quantify the angular distribution of f-actin fibers for cells in a given pattern as well as the overall collagen alignment from the SHG images.

To stain for focal adhesions, the cells were incubated with an anti-vinculin primary antibody (VIIF9 (7F9), mab 3574, Sigma-Aldrich, St. Louis, MO, USA) overnight at 4 °C, followed by incubation with a Texas Red secondary antibody (Mouse IgG (H+L), T862 1/EA, Invitrogen). Two-photon excited immunofluorescence images were collected using a 40× 0.8NA objective. This was done for both IOSE and OVCA433 cells with 20 cells analyzed for each scaffold. The number of focal adhesions per cell and integrated areas (following background subtraction) were determined in ImageJ.

For cadherin staining, the cells were incubated with an anti-E-cadherin (mouse, ab1416, Abcam) and anti-N-cadherin (rabbit, ab18203, Abcam, Cambridge, UK) primary antibody (at 1:200 dilution) overnight at 4 °C, followed by incubation with Alexa Fluor 488 (goat anti-rabbit IgG (H&L), ab150077, Abcam) and Alexa Fluor 594 (goat anti-mouse IgG (H&L), ab150116, Abcam) secondary antibody, respectively, for 1 h at room temperature. Fluorescent images of each respective channels were collected using a 40× 0.75NA objective. This was done for both IOSE and OVCA433 cells with 30 cells analyzed for each scaffold. Corrected total cell fluorescence (CTCF) was determined using ImageJ by measuring the integrated staining density and subtracting the total background.

### 2.6. Statistical Analysis

Statistical analyses of migration data, cell shape data, focal adhesion, and cadherin staining were performed in Origin 2017 (OriginLab, Northampton, MA, USA) first using ANOVA, followed by two-sample t-test analysis. Watson’s U^2^ tests were performed on f-actin and collagen fiber distributions using Oriana (Kovach Computing Services, Pentraeth, UK) to calculate directional statistics of the distribution and mean direction. Pearson correlation coefficients between these distributions were also calculated to measure correlation of the stress fibers and the collagen fibers in the stromal models.

## 3. Results

### 3.1. SHG Image-Based Blueprints for Fabrication

**Figure 1:**
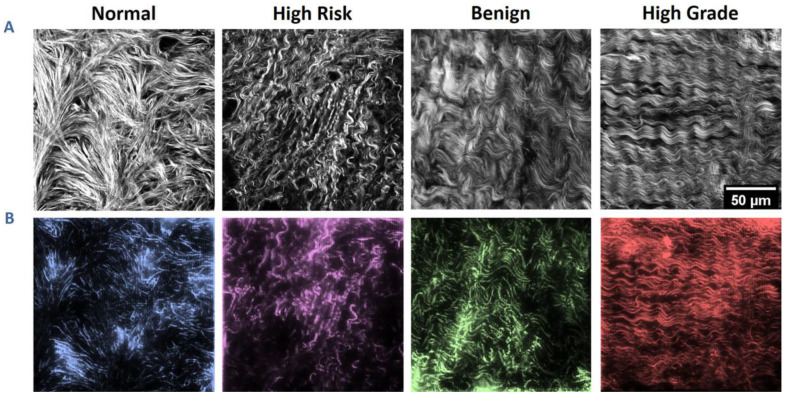
Ovarian stromal images and corresponding fabricated scaffolds. (**A**) Second-Harmonic Generation (SHG) optical sections of collagen from the four categories of ovarian tissues. (**B**) Two-photon excited fluorescence images of the resulting respective scaffolds. Each pattern is 200 × 200 µm in size with 10 µm in height. Scale bar = 50 µm.

To serve as blueprints for the scaffolds, we began with SHG images we previously collected and analyzed from normal ovarian tissues, high-risk tissues, benign tumors, and high-grade tumors, where these originated ~10 µm below the surface epithelium [23,24,25]. For statistical relevance, four images from each group were used in this study, where these were chosen at random from those properly classified by machine learning [25]. Figure 1A shows a representative SHG image of the collagen topography from each of the four groups. In general, the normal stroma has a mesh-like morphology with straight fibers, whereas the other tissues have varying degrees of alignment and periodicity [45].

As fibers can overlap with the focal volume, we used image processing techniques (e.g., eigenvalues of the Hessian matrix, thresholding, and tubeness) to discretize fiber structures from the SHG images, where the resulting images were used as the design templates. Figure 1B shows the resulting fabricated structures, where the scaffolds were stained with rhodamine B for contrast and imaged via two-photon excited fluorescence. Fidelity of the fabricated structures relative to the discretized model was 90% or higher to their respective template, where this was obtained by co-localization of both the spatial pixel overlap and the respective grayscale intensities between the model of the image data and the fabricated structure [47]. Immunofluorescence confirmed that Col I was incorporated in the GelMA + Col I scaffolds [46]. For the studies to follow, the scaffolds comprised 3 × 3 repeats of the same 200 × 200 × 10 µm pattern, for an overall size of 600 × 600 µm to yield sufficient area to simultaneously monitor multiple cells.

### 3.2. Cell Migration on Image-Based Scaffolds

**Figure 2:**
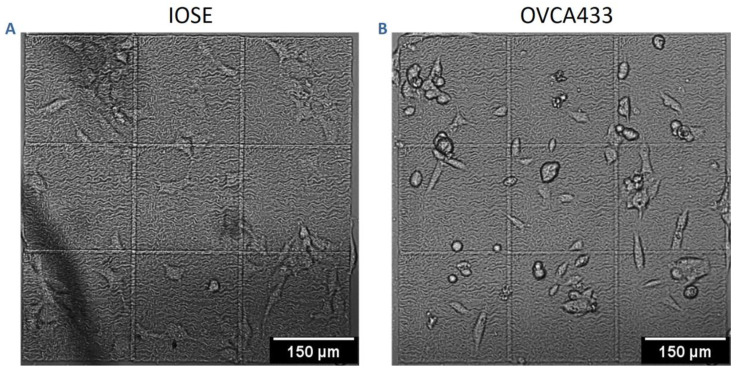
Phase-contrast images of IOSE (**A**) and OVCA433 (**B**) cells on a high-grade pattern. Scale bar = 150 µm.

We investigated how the highly different collagen topographic patterns in the four classes influenced cell migration dynamics. In order to decouple the cell and matrix contributions, we used three cell lines of varying metastatic potential—IOSE, OVCA433, and HEY—where the first represents a “normal” immortalized ovarian surface epithelial cell, and the latter two are moderately and highly metastatic ovarian cancer cells, respectively. Figure 2 shows representative phase contrast images of IOSE (Figure 2A) and OVCA433 (Figure 2B) cells on a high-grade pattern. Migration on the scaffolds was measured for 72 h, and trajectories were mapped as described in the methods.

**Figure 3:**
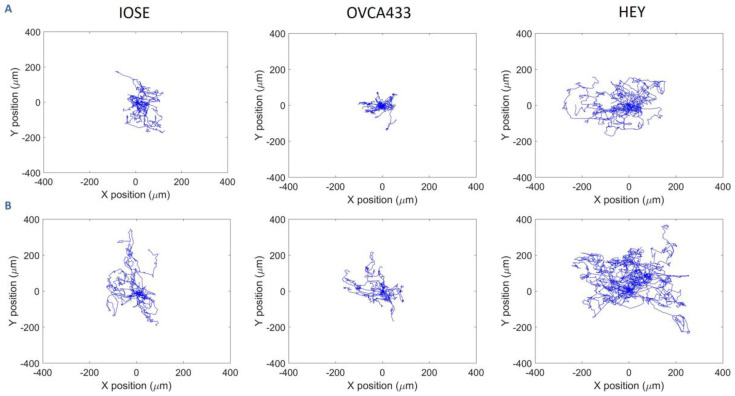
Representative trajectories of ovarian cell lines on image-based normal (**A**) and HGSOC (**B**) stromal models over 72 h. There were approximately 20 cells tracked in each case. Trajectories for these cells on the benign and high-risk models are not shown for simplicity.

Figure 3 shows representative migration trajectories of IOSE, OVCA433, and HEY cells (~20 in each case) on normal (Figure 3A) and high-grade (Figure 3B) models, respectively. For each cell type, we observed longer trajectories on the high-grade model relative to the normal stroma. For example, the majority of the IOSE trajectories on the latter were localized within 5 µm, where these ranged up to 200 µm on the cancer model. The cancer cells followed the same trend, although we note that the HEY cells were highly migratory in comparison to IOSE and OVCA433 lines, and the former had longer track lengths on the respective model. This agrees with our prior work studying migration of these cells on crosslinked gradients [57]. These results indicate that for all cell types, the highly aligned fibers promote cell migration to a larger extent than the more random structure provided by normal stroma.

**Figure 4:**
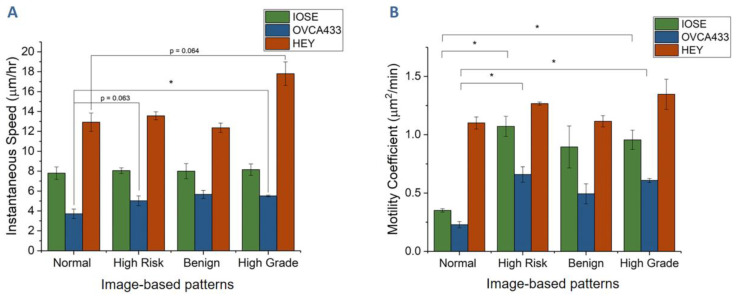
Migration dynamics for the three cell lines on scaffolds representing the four tissue types. (**A**) Instantaneous cell migration speed. (**B**) Motility coefficients. Migration was tracked over 72 h (* *p* < 0.05).

To quantify all trajectory data, we first determined the instantaneous velocity and motility coefficients. Figure 4A shows the average velocity values for the three cell lines on the four scaffolds. First, we note that the highly metastatic HEY cells were the fastest on each scaffold, which was consistent with our previous data using simple linear models [45,58]. In addition, we note that instantaneous speed did not change significantly for each of the cell lines on the different morphologies, with the only differences lying between the mesh-like normal and highly aligned high-grade models.

We next examined the role of motility, i.e., the ability to migrate in one direction before changing direction, where this was determined through measuring the mean square displacement (MSD; see Equation (1), Methods Section 2.4). The averaged motility results for the three cell lines on the four structures are shown in Figure 4B. First, we can compare the cell behavior on the different morphologies. The highly polar HEY cells showed the highest motility in comparison to the other cell lines, but these also had the weakest scaffold dependence. When comparing the role of the collagen morphology, we found the lowest motilities on the normal stromal model, which had a fairly random alignment. In contrast, the more aligned fibers of the high-risk and high-grade models resulted in higher motility for each cell line. In sum, these measurements showed that both the cell phenotype and fiber architecture were important factors in the resulting migration, although the different cells followed the same overall trends on the same scaffold.

### 3.3. Collagen Fiber Alignment Drives Cell Morphology

We next examined cell shape on the four classes of image-based models to determine how the collagen architecture affects the resulting phenotype. We quantified the cell morphology using circularity measurements. This metric is given by $\frac{4\pi A}{2p^{2}}$, where *A* and *p* are the area and perimeter, respectively, and lower values correspond to more elliptical shapes (more aligned), respectively.

**Figure 5:**
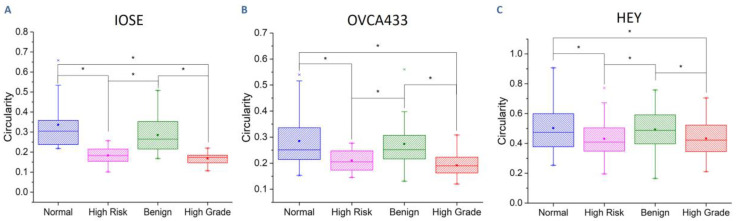
Cell circularity for IOSE (**A**), OVCA433 (**B**), and HEY (**C**) cells on image-based patterns (* *p* < 0.05).

Figure 5 gives the circularity for the three cell lines on the four image-based structures. We found the same trends in each case, where, for example, the cells were the most aligned on the high-grade model (i.e., lowest circularity) and least on the normal stroma. Cells on the high-risk model had similar circularities to those on the high-grade model. We note that both these models shown in Figure 1 also had highly aligned collagen fibers. Similarly, cells on the normal and benign tumor models had similar circularities, consistent with the comparable random fibers from the SHG images (although the benign tumor had thicker, more fibrotic like fibers). Overall, the OVCA433 cells had lower values of circularity than the IOSE, which likely was due to the more polar initial cell shape. The absolute values for the HEY cells were higher than the other cells types. As these cells showed the least scaffold-dependent motility, lower alignment may be expected. In sum, while the absolute values of the circularity varied between the cell types, the overall trends with significant differences for the four stroma models were the same in each case, where these are related to fiber alignment in the models.

### 3.4. Fiber Alignment Governs Cytoskeletal Expression and Alignment

**Figure 6:**
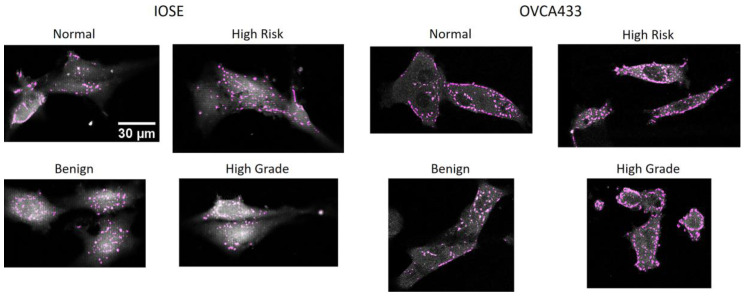
Representative two-photon excited immunofluorescence images of IOSE and OVCA433 cells stained with a primary anti-vinculin antibody and secondary antibody conjugated with Texas Red. Scale bar = 30 µm.

We next measured focal adhesion density on image-based structures to determine the extent that aligned fibers promoted enhanced expression. Here, we specifically stained for vinculin as it is a membrane-associated protein component of integrin-mediated adhesions that connects the cytoskeleton to the ECM [59,60]. Representative immunofluorescence (anti-vinculin) images for IOSE and OVCA433 cells are shown in Figure 6. These studies could not be conducted with HEY cells as they do not form discrete focal adhesions and the vinculin staining is diffuse [44].

**Figure 7:**
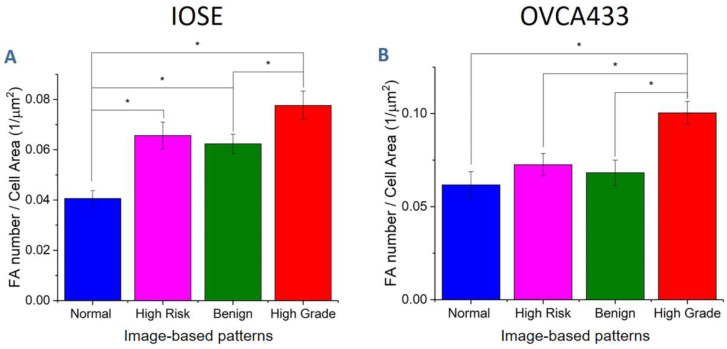
Focal adhesion density of IOSE (**A**) and OVCA433 (**B**) cells on image-based structures (* *p* < 0.05).

We quantified the focal adhesion density in terms of number of adhesions per cell area, and the results are shown in Figure 7. For the IOSE cells (Figure 7A), the focal adhesion expression was significantly higher on high-grade structures in comparison to the other scaffolds. These results suggest that the aligned crimping pattern promotes focal adhesion formation. In contrast, the expression was lowest on the linear, mesh-like fibers in the normal model. The OVCA433 cells (Figure 7B) showed very similar behavior, where these results indicated the matrix morphology was the dominant factor in the cell response, rather than the initial cell type.

**Figure 8:**
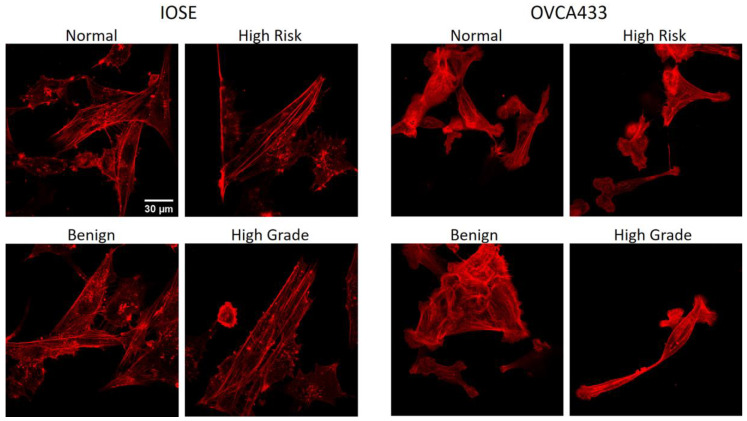
Two-photon excited fluorescence of f-actin filaments of IOSE and OVCA433 cells stained with Texas Red (phalloidin) on image-based patterns. Scale bar = 30 µm.

We next analyzed the spatial distribution of cellular stress fibers relative to the collagen morphology of the different stromal models. Representative images of fluorescence images (phalloidin) for IOSE and OVCA433 cells are shown in Figure 8. These studies could not be conducted with HEY cells as the actin filaments form only shorter segments, compromising the analysis.

We quantified the alignment of stress fibers and collagen fibers through Pearson correlation coefficients between the respective radial distributions (Table 1). Both cells types showed similar responses on the same respective matrix. Specifically, the highest correlations were on the high-grade (highest) and high-risk structures, and the lowest was on the benign model. Therefore, we conclude the highly aligned stromal architectures of the former promote stress fiber alignment. These results are consistent with the migration (Figure 4B) and cell shape (Figure 5) data, where both cells displayed higher motility on the high-grade models, as well as the lowest values of circularity (higher alignment). Lastly, we used the Watson’s U^2^ test to examine the distribution of the stress fibers for each cell/scaffold combination. For each cell type, the cells on the high-grade and normal models had the narrowest and broadest distribution, respectively. In analogy to the focal adhesion expression, these results indicate the matrix morphology drives the actin cytoskeleton characteristics of distribution width and alignment with respect to the collagen fibers.

**Figure 9:**
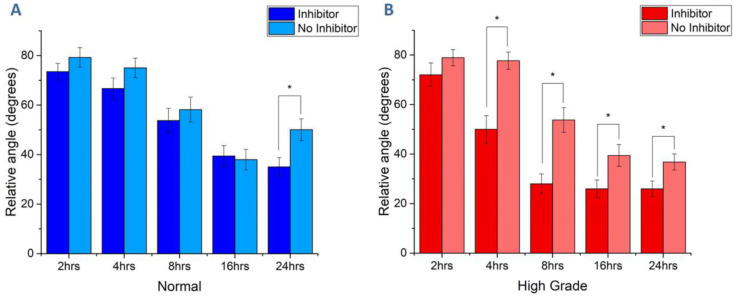
OVCA433 cell alignment on normal (**A**) and high-grade (**B**) stromal models in response to ROCK inhibition. Lower angles correspond to higher alignment, and significant differences were found at all time points after 2 h on the high-grade matrix. (* *p* < 0.05).

To begin to investigate the mechanism of the shape/cytoskeleton changes, we performed cell shape analysis following Rho-associated protein kinase (ROCK) inhibition (iY27632 at 10 µm). This treatment should result in further spreading and polarization as ROCK itself enhances contraction. To quantify the elongation, we measured the angle of the cells relative to the fiber axis, where lower angles correspond to increased alignment [61]. As we found the largest differences in motility, cell shape, and cytoskeleton expression/alignment for cells on the normal and high-grade models, we chose these scaffolds for this analysis. The alignment data for OVCA433 cells on normal and high-grade matrix are shown in Figure 9A,B, respectively (~*n* = 40 cells/scaffold). The only significant shape change in response to ROCK inhibition for cells on the normal matrix was after 24 h of adhesion, where increased alignment was observed. The data on the high-grade matrix are in strong contrast, where increased alignment (lower angle) was observed at all timepoints after 2 h. This increase may be due to an underlying contact guidance mechanism provided by the aligned, wavy fibers presented by this model. This would be consistent with both increased focal adhesion density and stress fiber alignment relative to the fibers.

### 3.5. Matrix Morphology Determines Cadherin Expression

**Figure 10:**
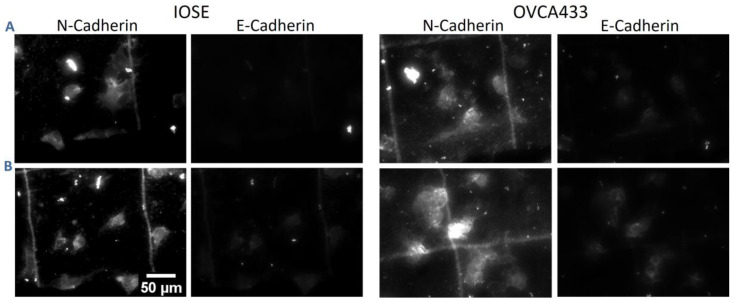
Representative immunofluorescence images of IOSE and OVCA433 cells stained with primary anti-E-cadherin and anti-N-cadherin antibodies and a secondary antibody conjugated with Alexa Fluor 488 and Alexa Fluor 594, respectively. (**A**) Normal and (**B**) high-grade models. Scale bar = 50 µm.

Modulation of both E-cadherin and N-cadherin expression has been reported in HGSOC [62,63,64,65]. Here, we quantified their relative scaffold-dependent expression to determine if/how these are influenced by the stromal morphology. Representative immunofluorescence images for IOSE and OVCA433 cells are shown in Figure 10. HEY cells exhibit negligible E-cadherin expression and were not used for the analysis. For quantification of the relative expression of individual cells on each scaffold, we measured the immunofluorescence intensity ratio relative to cells off the patterns (normalized to the same area; ~*n* = 30 cells/scaffold).

**Figure 11:**
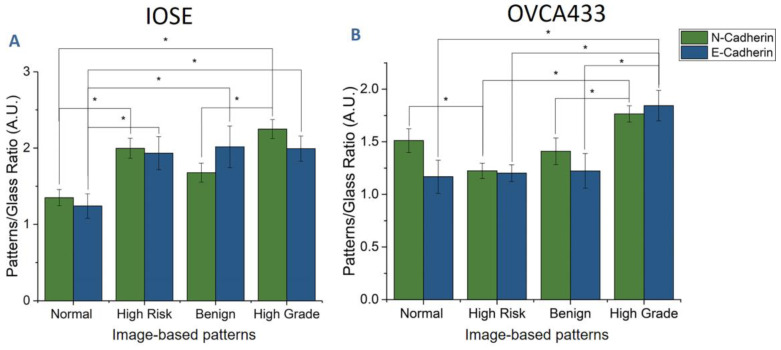
Immunofluorescence intensity ratio for cells on/off patterns of both E-cadherin and N-cadherin of IOSE (**A**) and OVCA433 (**B**) cells on image-based patterns (* *p* < 0.05).

Figure 11A shows the relative immunofluorescence intensity ratios of both E-cadherin and N-cadherin for IOSE cells on image-based patterns. The E-cadherin expression was significantly lower on the normal pattern in comparison to the other models, indicating that the more aligned collagen fiber architecture of the benign, high-risk, and high-grade scaffolds promotes its expression. Overall, the N-cadherin expression on the respective scaffold mirrored that of E-cadherin. Similar trends were found for the OVCA433 cells (Figure 11B); however, the largest differences were found between cells on the high-grade model relative to the rest, which were mostly not statistically different. This is similar to the motility results shown in Figure 4B, where the IOSE cells had a stronger scaffold-dependent motility than the OVCA443 cells.

## 4. Discussion

A deep understanding of the cell–stromal interactions in ovarian cancer is critical to the development of better diagnostics as well as assessments of treatment efficacy. This is a critical issue for high-grade disease as it can metastasize via exfoliation from the surface epithelium while lesions are still small [8,9,10,11]. However, studies of cell migration have been limited by the lack of biomimetic models [66]. While the role of collagen alignment in cancer (especially breast cancer) has been studied with different methods [31,67,68,69], it has not been possible to reproduce all aspects of the complex collagen architecture, specifically the fiber lengths, widths, and packing into the overall stromal morphology. The MPE fabrication approach is well matched to this task as we can exploit the freeform nature to create image-based scaffolds of different classes of tissues. Moreover, the cell–matrix interactions can be studied with cells of differing characteristics. As the ovary can be either the primary site or first metastatic site (from the fallopian tubes) in this cancer [70], we chose to model the surface of the ovary for these studies. We focused the analyses on migration and migration-related structural aspects (cell morphology, focal adhesion expression, and f-actin fiber alignment) as these processes are highly mis-regulated in ovary relative to normal tissues [45,46,57].

The primary finding of our study is that increased fiber alignment and crimping morphology of the high-grade stroma models enhance motility for all cell lines. In addition, the overall trends in the other migration-based metrics were similar for the normal IOSE and cancer cell lines on the respective four scaffolds. Specifically, the motility, cell alignment, f-actin alignment, and focal adhesion expression were highest on the highly aligned high-grade model and the lowest on the random mesh-like normal stromal models. Collectively, the similarity in response for a normal ovarian line and high-grade ovarian cancer cell lines on the same respective scaffold indicate the dynamics are governed to larger extent by the matrix morphology than the initial cell type. We note that there are some differences in absolute values in the migration-related metrics between the cell lines. For example, the IOSE cells showed the largest decrease in circularity on the aligned structures. This may be due to the larger cell size, which can interact with more fibers at the same time. This is analogous to prior work by Yamada, where they showed greater elongation of fibroblasts on closely packed 1D lithographic stripes than those further apart [71]. Overall, the OVCA433 cells had lower values of circularity, which likely is due to the more polar initial cell shape. The circularity values for the HEY cells were higher, where this may be attributed to the smaller cell size interacting with fewer fibers. Moreover, these cells show a larger distribution of cell shapes, which likely is due to their fast migration without organized stress fibers and focal adhesions.

One second commonality in findings is that the highly metastatic HEY cells had the fastest migration and highest motility, and these properties were only weakly substrate dependent. This may be due to these cells having already undergone an epithelial-to-mesenchymal transition (EMT) and migrate with a different mechanism than the IOSE or OVC433 cells. For example, these cells do not express discrete stress fibers or focal adhesions. We had also observed these differences using crosslinked concentration gradients [57].

We can discuss the putative migration mechanism in terms of contact guidance, where this process describes cell migration in response to anisotropic physical features of the ECM [72,73]. For example, cells can elongate and migrate when sensing local changes in substrate concentration or morphology. Contact guidance has been investigated in terms of ROCK signaling, where this well-known serine-threonine kinase acts on the cytoskeleton, regulating cell shape and migration via actomyosin contractility [74]. Importantly, this specific mechanism has been reported to play an important role in cancer metastasis [75]. For example, using ROCK inhibition, Provenzano et al. showed that an aligned collagen matrix (self-assembled gel model) provided contact guidance cues that significantly enhanced cell motility [73].

In our work, we drew upon such previous reports and performed a ROCK inhibition study to assess if contact guidance was implicated in the greater motility of ovarian cancer cells seeded on high-grade scaffolds (Figure 4). In Figure 9, we showed that the relative alignment of OVCA433 cells subjected to ROCK inhibition (reducing actomyosin contractility) was more influenced by the more highly aligned collagen fibers of the high-grade scaffolds in comparison to the normal model. Moreover, the crimping pattern in the former provides additional anisotropy and should further promote contact guidance, which is further consistent with both increased focal adhesion density (Figure 7) and stress fiber alignment (Table 1). 

We can also relate the relative N- and E-cadherin expression on these models to that observed in previous studies of ovarian cancer progression [63,76]. The cadherin extracellular domain provides cell–cell signaling, while the intracellular domain connects with the actin cytoskeleton by associating with catenins [77]. We observed that the relative expression of both E- and N-cadherin was significantly higher for IOSE and OVCA433 cells on the high-grade stromal model relative to the normal. While an EMT switch has been documented in many epithelial cancers, including ovarian, increased E-cadherin has also been associated with disease progression in HGSOC [63,76,78]. Relatedly, up-regulation of N-cadherin has been shown to promote cellular migration and increase motility [79,80,81]. These literature findings are consistent with our results on motility (Figure 4) and N-cadherin expression (Figure 11) for cells on the high-grade model. It is important to note that we are comparing the respective expression of each cadherin on the four tissue scaffolds, but not to each other, and thus we are not quantifying an EMT switch. Lastly, we performed staining for β-catenin and found a slight increase in expression for cells on the high-grade model, but the differences were not significant.

While we performed this detailed study on migration in ovarian cancer, it is insightful to compare the findings to those reported in the better studied breast cancer. For the latter, it has been shown that changes in collagen architecture (specifically aligned fibers) enhanced the motility of cancer cells in vivo and ex vivo [31,82,83,84,85,86]. For example, breast cancer cells on aligned collagen in microchannel models displayed enhanced and persistent migration [87,88,89,90]. Additionally, using a breast xenograft model, Condeelis found that cancer cells on parallel collagen fibers displayed highly directed migration [86]. We point out that normal breast stroma is mostly characterized by wavy fibers that become straighter in invasive cancer [91]. However, the shape in ovarian cancer is in the opposite direction, and we found greater motility for all cell lines on wavy fibers (high-grade model) over straight fibers of normal tissue. Thus, because the form of collagen remodeling may be different between cancer types, biomimetic models are needed to decouple the respective cell and matrix morphology contributions to the migration dynamics. We further suggest the MPE fabrication approach is broadly applicable to studying this class of problems across disease states.

## 5. Conclusions

Using multiphoton excited fabrication, we constructed image-based models of ovarian tissues. This technique is superior to other fabrication methods as the complex morphology of the collagen visualized by SHG microscopy can be recapitulated with high fidelity. Moreover, this approach affords hypothesis testing of respective cell and matrix morphology contributions to the migration dynamics. The key finding is that cell characteristics such as motility, cell shape, f-actin alignment, focal adhesion expression, and cadherin expression are mainly determined by the collagen fiber morphology to a larger extent than the initial cell type. Specifically, while the absolute values for these metrics were different for the three cell lines, the overall trends in the relative response to each scaffold were similar. Notably, we found enhanced motility and cell/cytoskeletal alignment on the highly aligned high-grade model. Conversely, the weakest corresponding responses were observed on the more random mesh-like normal stromal matrix. We suggest the scaffolds can be used for further cell biological studies and as platforms for testing of drug efficacy.

## Figures and Tables

**Table 1:** Pearson correlation coefficients between cellular f-actin fibers and stromal collagen fiber distributions of IOSE and OVCA433 cells for the four models.

Cell Line	Normal	High Risk	Benign	High Grade
IOSE	0.37	0.83	0.32	0.97
OVCA433	0.59	0.76	0.16	0.78
